# Koch on Malaria

**Published:** 1899-10-21

**Authors:** 


### KOCH ON MALARIA.

In his report on malaria, Professor Koch points out that, so far as his recent researches into the natural history of the malaria parasite go, this organism exists only in man and in certain forms of mosquito which are met with only in the summer. As is now well known, the mosquitos convey the malaria germ from one human being to another; but during a considerable portion of the year the mosquitos engaged in this transaction do not exist, or, at least, are inactive, and thus the disease would not arise again during the succeeding summer were it not that it is, so to say, carried over the winter by relapsing cases of malaria in man. These cases form the connecting link between one fever season and another. If no relapse occurred in any of the cases of malaria in a given district, it may be presumed that the mosquitos would find no germs—no malaria-charged blood—there in the beginning of the summer; and, so far as that district was concerned, malaria would become extinct until it was imported afresh. If, then, by the use of quinine relapsing cases could be cured and their blood freed from malaria parasites, or, we may add, if the access of mosquitos to such cases could be prevented by the use of curtains and other measures, it ought to be possible to put a check to the spread of the disease. Major Ross is engaged in proving that but one kind of mosquito is engaged in the spread of malaria, and that if this creature can be exterminated the disease will be put an end to. But if it be a fact that the whole life cycle of the malaria parasite lies exclusively between man and mosquito, and that no other form of animal is engaged in it, then it ought to be as easy—perhaps more easy—to break up the vicious circle of events on the human side by isolation and quinine, as it would be on the side of the mosquito by the use of kerosene and puddle drainage.

